# Targeted HIV testing for male partners of HIV-positive pregnant women in a high prevalence setting in Nigeria

**DOI:** 10.1371/journal.pone.0211022

**Published:** 2019-01-30

**Authors:** Semiu Olatunde Gbadamosi, Ijeoma Uchenna Itanyi, William Nii Ayitey Menson, John Olajide Olawepo, Tamara Bruno, Amaka Grace Ogidi, Dina V. Patel, John Okpanachi Oko, Chima Ariel Onoka, Echezona Edozie Ezeanolue

**Affiliations:** 1 Florida International University Robert Stempel College of Public Health & Social Work, Miami, FL, United States of America; 2 Department of Community Medicine, College of Medicine, University of Nigeria, Nsukka, Enugu, Nigeria; 3 Independent Researcher, Las Vegas, NV, United States of America; 4 School of Community Health Sciences, University of Nevada-Las Vegas, Las Vegas, NV, United States of America; 5 Research Resource Center, College of Medicine, University of Nigeria, Nsukka, Enugu, Nigeria; 6 HealthySunrise Foundation, Las Vegas, NV, United States of America; 7 Caritas Nigeria, Abuja, Federal Capital Territory, Nigeria; 8 Department of Pediatrics and Child Health, College of Medicine, University of Nigeria, Nsukka, Enugu, Nigeria; Agencia de Salut Publica de Barcelona, SPAIN

## Abstract

**Background:**

Partner HIV testing during pregnancy has remained abysmally low in sub-Saharan Africa, particularly in Nigeria. Males rarely attend antenatal clinics with their female partners, limiting the few opportunities available to offer them HIV testing. In this study, we evaluated the scale-up of the Healthy Beginning Initiative (HBI), a community-driven evidenced-based intervention to increase HIV testing among pregnant women and their male partners. Our objectives were to determine the: (1) male partner participation rate; (2) prevalence of HIV among male partners of pregnant women; (3) factors associated with HIV positivity among male partners of HIV-positive pregnant women.

**Methods:**

We reviewed program data of expectant parents enrolled in HBI in Benue State, north-central Nigeria. During HBI, trained lay health workers provided educational and counseling sessions, and offered free onsite integrated testing for HIV, hepatitis B virus and sickle cell genotype to pregnant women and their male partners who participated in incentivized, church-organized baby showers. Each participant completed an interviewer-administered questionnaire on demographics, lifestyle habits, and HIV testing history. Chi-square test was used to compare the characteristics of HIV-positive and HIV-negative male partners. Simple and multivariable logistic regression models were used to determine the association between participants' characteristics and HIV positivity among male partners of HIV-positive women.

**Results:**

Male partner participation rate was 57% (5264/9231). Overall HIV prevalence was 6.1% (891/14495) with significantly higher rates in women (7.4%, 681/9231) compared to men (4.0%, 210/5264). Among the 681 HIV-positive women, 289 male partners received HIV testing; 37.7% (109/289) were found to be HIV-positive. In multivariate analysis, older age (adjusted odds ratio [aOR]: 2.45, 95% confidence interval [CI]: 1.27–4.72 for age 30–39 years vs. <30 years; aOR: 2.39, CI: 1.18–4.82 for age ≥40 years vs. <30 years) and self-reported daily alcohol intake (vs. never (aOR: 0.35, CI: 0.13–0.96)) were associated with HIV positivity in male partners of HIV-positive women.

**Conclusion:**

The community-based congregational approach is a potential strategy to increase male partner HIV testing towards achieving the UNAIDS goal of 90% HIV screening. Targeting male partners of HIV-positive women for screening may provide a higher yield of HIV diagnosis and the opportunity to engage known positives in care in this population.

## Introduction

HIV testing services (HTS) is recognized as a critical gateway towards achieving epidemic control and meeting the goal of the HIV care cascade promptly[1]. Despite concerted efforts to expand HTS in Nigeria, coverage has consistently remained low among men. The Government of Nigeria estimates a 23.5% HIV testing coverage in the male population[2]. In 2016, among men with new HIV diagnosis in Nigeria, 41% of them received HIV testing in the advanced stage of the disease[3]. Low rates of testing and late HIV diagnosis in men have contributed to high mortality with an estimated 81,000 male deaths attributed to the disease in 2016[3].

The antenatal period presents an opportunity to engage male partners of pregnant women in HTS and promote healthy sexual behavior especially in couples in discordant partnerships. Studies highlight that during this period, there is increased HIV infectivity in affected females[4,5] and HIV transmission risks to their uninfected male partners[5]. Recognizing the unmet need for HTS among couples, current guidelines by the Federal Ministry of Health of Nigeria recommend implementing partner testing strategies across community and facility HTS delivery models[6]. One of such strategies is offering couple HIV testing during routine antenatal care (ANC) visits that has been documented to improve disclosure, sexual decision-making[7,8], and maternal and child health outcomes in the prevention of mother-to-child transmission (PMTCT) interventions[9–12]. However, available data show that 36% of pregnant women in Nigeria do not present for ANC[13], and male partner attendance during ANC visits is low[14,15], limiting the few opportunities available to offer men HIV testing. For example, a recently published retrospective analysis of PMTCT data of 11.8 million pregnant women collected over a five-year period in Nigeria found that only 2.2% of male partners received an HIV test during an ANC visit[15]. To address this gap in testing coverage, novel approaches that promote couple HIV testing and increase HIV case finding efficiency are urgently needed. Community-based interventions that address barriers to facility-based HTS may offer an opportunity to achieve high testing coverage and identify undiagnosed HIV infection in male partners by offering HTS closer to where they reside[16–20].

As reported elsewhere[21–23], our team previously conducted a cluster-randomized trial using a community-based intervention that significantly increased male partner HIV testing rates in southeast Nigeria. The Healthy Beginning Initiative (HBI) trial showed significantly higher HIV testing rates among male partners in the intervention group compared to the control group (84% vs. 34%, p < 0.001)[24]. Our next challenge as a team of academics and HIV program implementers was to scale-up the effective community-based HBI intervention, beyond previous trial sites, to communities in Benue State, north-central Nigeria—the state with the highest prevalence of HIV in the country according to sentinel surveys[25]. In this paper, we report our findings on (1) the male partner participation rate; (2) the prevalence of HIV among male partners of pregnant women; (3) and factors associated with HIV positivity among male partners of HIV-positive pregnant women, in the community-based HBI intervention.

## Materials and methods

### Study design, population and setting

We reviewed program data from a cohort of 9231 self-identified pregnant women and their male partners participating in the HBI from July 2016 to August 2017. HBI was implemented by Caritas Nigeria, a local President's Emergency Plan for AIDS Relief (PEPFAR) implementing partner and conducted in 80 churches across 12 local governments in Benue State, north-central Nigeria. Benue State has a land mass of 30,955 sq. Km with abundant arable land and an estimated population of five million[26]. In 2014, the HIV prevalence estimate in the state was 15.4%[25], and only 60% of pregnant females received antenatal care.[27]

### Description of the Healthy Beginning Initiative

A detailed description of the program has been published previously[21]. HBI was designed as a sustainable, culturally adapted community-driven program delivered by trained lay health workers residing within the community to identify pregnant females, implement health interventions and support linkage to health services for women and their families. Briefly, it has three main platforms: *Prayer sessions* during church services are used to identify pregnant women through an announcement that was delivered by a priest. Each Sunday, the priest asked pregnant women and their male partners in the congregation to approach the altar for a prayer. He prayed for a healthy pregnancy, successful delivery, and encouraged pregnant women to seek antenatal care at a health facility. Church-organized *baby showers* provided opportunities for interventions that included health education, counseling and free onsite integrated laboratory screening for HIV, hepatitis B virus, and sickle cell genotype that was delivered by trained lay health workers. This strategy replaced the HIV-only testing approach which may lead to stigma. Participating couples were provided an incentive in the form of a “Mama Pack”, a small gift which included essentials needed for a hospital or home birth and immediate postnatal periods. Health assessments such as weight, height, and blood pressure were also carried out. *Baby receptions* held 6–8 weeks after birth allowed for post-delivery follow-up and enhanced referral for early infant diagnosis for HIV-exposed infants. Additionally, missed male partners who were not present during the baby showers were offered HIV testing during the baby receptions. Caritas Nigeria program staff ensured HBI participants who tested positive for HIV received appropriate HIV prevention, treatment, and care services through referrals to health facilities within the state.

### Rapid HIV testing

According to the national guidelines on HTS, all HIV tests were performed onsite by trained lay health workers using Determine rapid HIV antibody tests (Abbott Laboratories, IL, US). A positive test result was subsequently confirmed using Uni-Gold (Trinity BioTech, ROI). If a discordance occurred between the Determine and Uni-Gold results, Stat-Pak (Inverness Medical—Biostar Inc., DE, US) rapid HIV antibody test was used as a “tie-breaker”.

### Data collection

Each participant had completed a structured questionnaire administered by a program staff. Socio-demographic information collected included sex, age, marital status, highest educational attainment, and distance to the nearest health facility. Participants were also asked if they had ever had an HIV test and their frequency of alcohol and tobacco consumption.

### Data analysis

Descriptive analyses were conducted to calculate the prevalence of HIV in both pregnant women and their male partners. Chi-square test was used to compare the characteristics of HIV-positive and HIV-negative male partners. Simple and multivariable logistic regression models were used to determine the participant characteristics associated with HIV positivity among male partners of HIV-positive women. The significance of odds ratios was determined with 95% confidence interval (CI). For all analyses, p-values of <0.05 were considered statistically significant. All analyses were performed using Stata 13 (College Station, Texas). We could not correctly identify partnership for 110 male partners. Therefore, a final matched sample of 5154 men and their 5154 pregnant female partners with complete HIV test information was used for the analysis of male partners.

### Ethical consideration

The HBI was a voluntary HIV testing program implemented by Caritas Nigeria. As such, individuals who participated were not consented. The Health Research Ethics Committee of the University of Nigeria Teaching Hospital, Enugu, Nigeria gave the approval to conduct a secondary data analysis of the de-identified HBI program data and publish the findings.

## Results

### Demographic characteristics

A total of 14495 individuals participated and were screened for HIV in the HBI from July 2016 to August 2017. Of the 9231 women who participated, 5264 had male partners who received HIV testing, a male participation rate of 57%. Demographic data for the individuals are shown in Table 1. Mean age ± standard deviation was 27.5 ± 8.3. Most participants were married (99.5%), and more than 60% had attained a secondary level education or higher. Slightly less than a quarter reported that they had never received an HIV test. Alcohol consumption was high, and tobacco use was relatively low at 34% and 11% respectively.

**Table 1:** Characteristics of female and male HBI participants in Benue State, Nigeria.

Variables	Frequency	Percentage
Age group		
<20	1402	19.7
20–29	8198	56.6
30–39	3681	25.4
40+	1214	8.4
Sex		
Female	9231	63.7
Male	5264	36.3
Marital status		
Married	14380	99.2
Unmarried	110	0.8
Missing	5	0.03
Educational level		
No formal education	1967	13.6
Primary education	3728	25.7
Secondary and above	8793	60.7
Missing	7	0.05
Self-reported previous HIV testing		
No	3266	22.5
Yes	11204	77.3
Missing	25	0.17
Alcohol use		
Never	9547	65.8
Occasionally	4216	29.1
Daily	683	4.7
Missing	55	0.4
Tobacco use		
Never	12846	89.4
Occasionally	384	2.7
Daily	1145	7.9
Missing	120	0.8
HIV status		
Positive	891	6.1
Negative	13604	93.9
TOTAL	14495	100

Percentages are approximated and may not add up to 100%.

### Prevalence of HIV among pregnant females and male partners

Table 2 shows the prevalence of HIV by sex among HBI participants. Overall HIV prevalence was 6.1% (7.4% among females and 4% among the male partners).

**Table 2:** HIV status of HBI participants in Benue State, Nigeria by sex.

HIV status	Sex		Total N (%)
	Female N (%)	Male N (%)	
Negative	8550 (92.6)	5054 (96.0)	13604 (93.9)
Positive	681 (7.4)	210 (4.0)	891 (6.1)
Total	9231 (100.0)	5264 (100.0)	14495 (100.0)

### Comparison of male partners’ characteristics by HIV status

Among the couples in our analyses, 4764 (92.4%) were concordant HIV-negative, and 390 were in a relationship where one or both partners tested HIV-positive. Of the 390 couples, 281 (72%) were in serodiscordant relationships with the female partner more likely to be the HIV infected partner compared to the male (180 vs. 101). Table 3 shows individual characteristics of the male partners and differences between them according to their HIV status. Male partners who had a confirmed positive test result for HIV were significantly more likely to be older, and have female partners who also tested positive for HIV.

**Table 3:** Comparison of characteristics of male partners of pregnant females in HBI in Benue State, Nigeria by HIV status.

Variables	HIV -	HIV +	p-value
	N (%)	N (%)	
Age group (years)			
<30	2017 (97.8)	46 (2.2)	0.000
30–39	1901 (95.4)	92 (4.6)	
40+	973 (93.1)	72 (6.9)	
Educational level			
No formal education	279 (96.9)	9 (3.1)	0.595
Primary education	905 (95.3)	45 (4.7)	
Secondary and above	3758 (96.0)	156 (4.0)	
Missing	2	0	
Ever tested for HIV			
No	1176 (96.6)	42 (3.5)	0.347
Yes	3757 (95.7)	168 (4.3)	
Missing	11	0	
Alcohol use			
Never	2019 (95.8)	89 (4.2)	0.674
Occasionally	2356 (95.9)	102 (4.2)	
Daily	553 (96.9)	18 (3.2)	
Missing	16	1	
Tobacco use			
Never	3659 (96.1)	148 (3.9)	0.488
Occasionally	280 (96.2)	11 (3.8)	
Daily	968 (95.1)	50 (4.9)	
Missing	37	1	
Female partner’s age			
<30	4091 (96.6)	145 (3.4)	0.000
30–39	778 (92.7)	61 (7.3)	
40+	75 (94.9)	4 (5.1)	
Female partner’s educational level			
No formal education	840 (95.9)	36 (4.1)	0.987
Primary education	1474 (96.0)	62 (4.0)	
Secondary and above	2627 (96.0)	112 (4.1)	
Missing	3	0	
Female partner HIV status			
Negative	4764 (97.9)	101 (2.1)	0.000
Positive	180 (62.3)	109 (37.7)	
TOTAL	4944	210	

### HIV positivity among male partners of HIV-positive females

Among the 681 women with HIV diagnosis, 289 male partners participated in HBI and received HIV testing; 37.7% (109/289) were found to be HIV-positive. Table 4 highlights the results of logistic regression to determine the factors associated with HIV positivity among male partners of these HIV-positive women. In bivariate analysis, age was the only variable significantly associated with having an HIV positive status. In multivariate analysis, the odds of a male partner having HIV diagnosis were higher in the 30 to 39-year (adjusted OR (aOR): 2.45, 95% CI: 1.27–4.72) and ≥40-year (aOR: 2.39, 95% CI: 1.18–4.82) age groups compared to those who were <30 years. Compared to those who self-reported that they never took alcohol, the odds of being HIV-positive were significantly lower if the participant self-reported daily alcohol intake (aOR: 0.35, 95% CI:0.13–0.96). After adjustment for potential confounders, no association was found between HIV positivity and educational level, ever tested for HIV, female partner’s age and educational level, and tobacco use.

**Table 4:** Simple and multivariable logistic regression analyses of factors associated with HIV positivity among male partners of HIV-positive pregnant females in Benue State, Nigeria (n = 289).

Variables	Crude OR (95% CI)	p-value	Adjusted OR (95% CI)	p-value
Age group (years)				
<30	REF		REF	
30–39	2.45 (1.27–4.72)	0.007	2.66 (1.33–5.33)	0.006
40+	2.39 (1.18–4.82)	0.016	2.26 (1.02–4.99)	0.043
Educational level				
No formal education	REF		REF	
Primary education	3.03 (0.89–10.43)	0.079	2.21 (0.59–8.34)	0.242
Secondary and above	1.84 (0.58–5.88)	0.302	1.33 (0.37–4.77)	0.659
Ever tested for HIV				
No	REF		REF	
Yes	1.77 (0.94–3.33)	0.077	1.52 (0.77–2.98)	0.225
Female partner’s age				
<30	REF		REF	
30–39	1.61 (0.95–2.73)	0.077	1.45 (0.74–2.47)	0.223
40+	0.37 (0.08–1.72)	0.202	0.41 (0.07–1.87)	0.285
Female partner’s educational level				
No formal education	REF		REF	
Primary education	1.30 (0.66–2.56)	0.440	1.34 (0.63–2.83)	0.450
Secondary and above	1.09 (0.59–2.03)	0.788	1.06 (0.52–2.15)	0.869
Alcohol use				
Never	REF		REF	
Occasionally	0.74 (0.45–1.22)	0.236	0.65 (0.38–1.13)	0.126
Daily	0.40 (0.15–1.07)	0.067	0.34 (0.12–0.95)	0.040
Tobacco use				
Never	REF		REF	
Occasionally	0.75 (0.29–1.93)	0.556	0.81 (0.29–2.27)	0.688
Daily	1.02 (0.57–1.83)	0.946	0.97 (0.51–1.87)	0.939

## Discussion

Findings from this study demonstrated that the scale-up of HBI was able to achieve a high male participation rate and that significant gender differences in the prevalence of HIV exist. Also, HIV seropositivity rates among male partners of HIV-positive pregnant women who participated in the HBI was about nine times that of the general male study population.

Male partner participation rate of 57% observed in this study was lower than the 84% reported in the HBI trial in Enugu State, southeastern Nigeria[24], but exceeds results from previous studies on recruiting male partners for HIV testing which ranged from 20% to 40.1%[19,28–32]. Thus, our findings support HBI as a potential strategy for attracting male partners to HTS towards achieving the United Nations (UN) target to identify 90% of undiagnosed HIV infection by 2020[1]. It is unclear why our findings are inconsistent with those of the HBI trial. The different cultural contexts in which HBI was conducted may offer a plausible explanation. Gender norms that have a strong influence on male partners' involvement in pregnancy-related events[33,34] may be more pronounced in this setting compared to the southeastern region of Nigeria. Future studies to understand the socio-cultural contexts that enhance male participation in HTS may be beneficial in designing culturally acceptable and scalable partner testing interventions. For example, a study in southern Nigeria incorporated local cultural norms and gender beliefs in designing a group talk intervention that was shown to be effective in improving male involvement in antenatal care[35].

Overall HIV prevalence of 6.1% in this study was higher than the national average of 2.9%[3] but lower than the estimated 15.2% in the sentinel survey in Benue state[25]. Sentinel surveys use facility-based HTS data that may overestimate the HIV prevalence in a population. It is likely that individuals with symptoms associated with HIV infection may more readily seek care at health facilities, be identified as at-risk patients by health workers and offered HIV testing. Therefore, the HIV prevalence found in this study may be more representative of the general population compared to the sentinel surveys.

HIV prevalence in men was notably lower than in their female counterparts. In sub-Saharan Africa, women are disproportionately affected by HIV compared to men[36]. The biological nature of women and associated risk factors in pregnancy have been shown to increase their susceptibility to HIV infection[4,5,37]. High fertility rates seen in this high HIV prevalence setting may further fuel the epidemic. Given the gender differences in HIV diagnosis among couples, there is a need to implement evidence-based strategies for HTS that are targeted at male partners to identify those at- risk early and increase case-finding yield for those with undiagnosed infection.

In our study, among couples where one or both partners tested HIV-positive, about 72% were in serodiscordant relationships; females were almost twice as likely to be the infected partner. These findings are in disagreement with other researchers that suggested equal transmission probability in both men and women[38,39]. Intuitively, one would expect high positive concordance rates among sexually active, stable unions; however, studies particularly in sub-Saharan Africa have shown wide variation in discordance in stable partnerships, ranging from 37% to 85% [39–41]. Chemaitelly et al. found that in countries with HIV prevalence of less than 10%, about 75% of partnerships affected by HIV are discordant[40]. Individual-level factors such as extramarital affairs[38], high plasma viral loads[39,40], young age[42] have been attributed to the high rates of discordance. Due to the substantial risk of HIV transmission in serodiscordant couples, the adoption of several HIV prevention strategies that can dramatically lower the risk within these relationships will be beneficial. It is essential that the infected partner be offered counseling and immediately commenced on antiretroviral therapy to reduce viral loads in body fluids to undetectable levels to prevent transmission. Another highly effective strategy is the use of pre-exposure prophylaxis (PrEP) by the HIV-negative partner. Accordingly, the World Health Organization[43] and the Government of Nigeria[6] recommend PrEP in combination with other HIV prevention methods as an additional intervention to prevent transmission to an uninfected partner. However, studies have shown that inconsistent and incorrect use of PrEP may reduce its effectiveness[44,45]. Therefore, it is important that counseling on risk-reduction strategies, adequate treatment preparation, support for adherence and engagement in PrEP services be made available to intending participants to mitigate challenges.

This study has several limitations. First, the HIV prevalence in men and seropositive concordance rates may be underestimated. It is likely that only men who had previously tested HIV negative participated and known HIV-infected male partners abstained from participating in HBI given their status. However, HBI was not an HIV-only testing program. Like we did in the initial study[21–23], HBI included other health assessments such as weight, height and blood pressure measurements, and tests for hepatitis B virus infection and sickle cell disease to negate the effects of the stigma associated with an HIV-only testing approach. During church announcements of the program, the priest–who is regarded as an authority, stressed the need to have regular health checks (not just HIV tests only) and encouraged men to accompany their pregnant partners to provide support. In addition, HBI provided incentives to women who participated with or without their male partner. We believe these factors could have had more impact on male partner participation rather than a previous HIV test result. Second, our findings may not be generalizable to other populations as this study was conducted in predominantly rural communities in the north-central region of Nigeria. Third, without phylogenetic analyses, the linkage between concordant couples, much less the potential directionality of transmission from an affected female to her male partner is impossible. Addressing the viral-linkage status is of utmost importance as was the case in the HPTN052 study. In this trial, researchers observed that 36% of the infections in the partners were unlinked to the index participant confirming that the transmission occurred outside the partnership[46]. Finally, because we relied on secondary data, we did not assess individual-level risk factors such as having multiple sexual partners and sexual network characteristics that may have a significant impact on HIV acquisition among couples.

## Conclusion

The community-based congregational approach is a potential strategy to increase male partner HIV testing towards achieving the UN goal of 90% for HIV screening. Targeting male partners of HIV-positive females for screening may provide a higher yield of HIV diagnosis and the opportunity to engage known positives in care in this population.

## Supporting information

S1 Dataset Supporting minimal dataset. (XLSX)
